# A pilot study on treatment content in virtual reality-assisted aggression therapy at a maximum-security forensic psychiatric clinic

**DOI:** 10.1038/s41598-025-01194-w

**Published:** 2025-05-16

**Authors:** Fredrik Sivermo, Fernando Renee González Moraga, Märta Wallinius

**Affiliations:** 1 Research Department, Regional Forensic Psychiatric Clinic, Växjö, Sweden; 2 Evidence-based Forensic Psychiatry, Department of Clinical Sciences Lund, Psychiatry, Lund University, Lund, Sweden; 3 Centre for Ethics, Law and Mental Health, Institute of Neuroscience and Physiology, The Sahlgrenska Academy, University of Gothenburg, Gothenburg, Sweden

**Keywords:** Virtual reality, VR, Aggression, Forensic psychiatry, VRAPT, Treatment, Content analysis, Psychology, Health care, Health services, Rehabilitation

## Abstract

Previous findings on results of treatment of aggression in violent offenders show inconsistent results, and implementations of such treatments have demonstrated varying success with sometimes marginal gains in forensic settings. New methods, incorporating virtual reality as a tool for experiential learning, have been put forward yet require deepened investigations concerning both treatment content and effects. The principal objective of this study is to examine the treatment content of the revised VRAPT intervention. Specifically, the study focuses on understanding how the content of the VRAPT intervention is conceptualized from the perspectives of both patients and therapists. Inductive manifest content analysis was applied on content of treatment workbooks (*N* = 6 + 7), provided by both patients and therapists as part of seven concluded VRAPT treatments at a maximum-security forensic psychiatric clinic in Sweden. Three manifest content categories were identified, relating to treatment content: Skills-training, Tailoring of the intervention, and Self-awareness. While generally quite similar, some potentially important differences between patients’ and therapists’ perspectives on the VRAPT intervention were apparent. The findings suggest the necessity of further research into optimizing VR-assisted treatments in forensic psychiatry.

## Introduction

Given the diversity of aspects influencing aggressive behaviours and the inconclusiveness of current empirical findings, the quest for a comprehensive and empirically supported model of aggression and its treatment continues^1–3^. Meanwhile, substantial knowledge on risk factors for general criminality, and violence specifically, is available^4–6^. However, risk-based models for understanding aggression may be overly simplified, since each risk factor may constitute an indispensable part of an unnecessary (i.e., it could just as well occur in its absence) yet collectively sufficient trigger of aggression^7^. When evaluated, structured risk assessments in general show a varying prediction of violence, with assessment models used for prediction of imminent aggression showing better prediction than longer-term models^8^. However, while such models can provide crucial information on possible risk factors, they do not provide direct guidance for the management of these risk factors that may be used in the treatment, or prevention, of aggression.

Studies on treatment of aggression show somewhat inconsistent yet promising results, favoring models applying cognitive behavioral treatment (CBT) in treatment of violent offenders^9^. When evaluating treatment interventions, however, their feasibility in the clinical practice they are designed for must be considered, and implementations of aggression treatment interventions in forensic settings have demonstrated varying success with sometimes only marginal gains^3^. Within the context of forensic psychiatry, where patients demonstrate complex clinical needs e.g., with a high comorbidity of mental disorders, antisocial cognitions, substance use, impulsivity and/or lack of empathy^10–13^, an effective implementation of treatment interventions seems crucial since these persons constitute a demanding but also vulnerable group, both in society and in clinical settings^14–16^. Aggression is a common problem in forensic psychiatric patients^17,18^ and is considered central to patient management^19,20^. However, the evidence-base for interventions in general in forensic psychiatry is scarce^21^, stressing the need for more research into development and evaluation of aggression treatments in forensic settings^1,22–24^.

In a recent effort, immersive virtual reality (VR) has been put forward as a potentially viable method for both assessment and treatment in general^25,26,23^, and of aggression in particular^1,27^. The argument advanced is that VR technologies, through potentially providing an ‘as-if’ experience, not merely addresses the gap between real life and clinical and/or research settings, but in so doing also might improve the prognostic value of associated psychological research^28,29^^30–32^. This is an important point, particularly in the context of forensic psychiatric populations, where cognitive deficits and paranoid ideations are common^29^. Such characteristics may limit the effectiveness or appropriateness of VR-based interventions for certain individuals. These limitations should be acknowledged when considering the broader applicability of VRAPT and highlight the importance of careful patient selection and adaptation of the intervention to individual needs.

The ‘as-if’ experience of VR technology is thought to derive from what has been described as the three core concepts of VR: immersion, presence, and embodiment^33–36^. Immersion means that the VR technology, admittedly to a varying degree^37^, provides an alternative environment substituting physical reality^38^. Presence is described as the user’s acceptance of, and physical, social as well as emotional engagement with, the virtual environment^39,40^. Finally, embodiment might be understood as emerging through the previous two: the incorporation of experiencing, acceptance of, and engagement with an immersive virtual environment that to a high enough degree shuts out physical reality. Subsequently, a sense of being in such an environment, of having agency and ownership constitute components of embodiment^41^, and are thus important parts in establishing a sense of “being” in a virtual environment^42^.

However, much remains to be investigated regarding VR-assisted treatment of aggression before any firmer conclusions on its feasibility and effectiveness can be drawn^17,43,44^. The first randomized controlled trial of the VR Game for Aggressive Impulse Management (VR-GAIME)^45^ failed to show any difference between VR-GAIME relative to the control condition^46^. Similarly, a randomized controlled trial investigating the Virtual Reality Aggression Prevention Training (VRAPT)^47^, although finding positive effects post-treatment on self-reported hostility, anger control and non-planning impulsiveness, did not demonstrate effects at follow-up^17^. Recently, a revised version of VRAPT^1^, in a small pilot study on imprisoned violent offenders, demonstrated positive effects on self-reported emotion regulation and aggression both post-intervention and at a 3-month follow-up^48^. When the same VRAPT version was evaluated within forensic psychiatric care, patients described foremostly positive experiences from VR-assisted role-plays, and less positive experiences in relation to motivation for aggression-focused treatment and technological limitations^27^. However, these initial studies do not in their own provide “evidence of absence” but should rather be interpreted as “absence of evidence” in this specific field, why continued research into the specific components of VR-assisted aggression treatments is needed.

The principal objective of this study is to examine the treatment content (e.g., treatment goals, session tasks, observations during sessions, homework activities) of the revised VRAPT intervention. Specifically, the study focuses on understanding how the content of the VRAPT intervention is conceptualized from the perspectives of both patients and therapists. It was expected that patients and therapists would conceptualize the content of the revised VRAPT intervention in ways that reflect distinct and identifiable themes. These themes were anticipated to relate to how the intervention supports emotional regulation, enhances self-awareness, and promotes behavioral change. The study aimed to explore and describe these thematic categories in-depth, based on treatment workbooks and participant perspectives.

## Materials & methods

This study was conducted at a maximum-security forensic psychiatric clinic in Sweden. Participants were recruited through clinical referrals, and only those meeting inclusion criteria (ongoing forensic psychiatric care, established pattern of violence, fluency in Swedish) were considered. Data were analyzed manually using inductive manifest content analysis without software support. All coding was initially conducted by the author (FS). However, the final categories and subcategories were refined and determined in consensus with last author MW. Subsequently, categories were interpreted, leading to a final narrative synthesis.

The current study applies inductive manifest content analysis^49^ on data from treatment workbooks, retrieved from a case-series pilot study including a diverse sample of forensic psychiatric patients with predominantly reactive aggression problems; exploring the content of virtual reality-assisted aggression treatment as defined in the manualized and recently revised VRAPT intervention^1^.

### Participants

#### Patients

Seven patients from a maximum-security forensic psychiatric clinic (6 males, 1 female; mean age 36 years old) were recruited as per the following inclusion criteria: (1) ongoing forensic psychiatric care, (2) established pattern of violence and current issues with reactive aggression, (3) having undergone VRAPT treatment. Criteria for exclusion consisted of (1) inability to speak and read Swedish, (2) epilepsy, (3) IQ < 70, (4) severe autism spectrum disorder, (5) acute psychotic state, (6) security considerations that prohibited participation. The composition of the sample was determined primarily by the willingness and availability of patients to participate in the study, rather than by a predetermined gender distribution. In high-security forensic psychiatric settings, participation in research is often limited due to clinical, legal, and motivational factors. The inclusion of one female participant reflects the actual population distribution at the clinic during the recruitment period and her expressed interest in participating. Regarding sample size, the decision to work with seven participants aligns with qualitative research guidelines suggesting that data saturation can be achieved with small, information-rich samples, particularly when working with a relatively homogeneous group and a focused research question. Nevertheless, we acknowledge the limitations of this sample size and encourage future studies with larger and more diverse cohorts.

#### Therapists

Therapists conducting VRAPT treatments were all, except one who at the time of the study was completing the final resident year, licensed psychologists or CBT therapists experienced in forensic psychiatry. Additionally, all underwent 16 h of specific training in the VRAPT methodology prior to treatment delivery.

## VRAPT intervention

This version of the VRAPT intervention is theoretically based in CBT and the General Aggression Model (GAM)^50–52^. Spanning over 16 sessions divided into four phases^1^, VRAPT begins with a general introduction to both the intervention in itself and the virtual environment. The second phase concentrates on skills training in assessing and discerning emotions – portrayed by the facial expressions of avatars within the virtual environment – together with practicing self-management of physiological reactions (as measured by e.g., variations in heart rate and skin conductance). In phase three, the patient practices interpersonal and problem-solving skills through therapist-led role plays in VR; the therapist actively participates in the virtual environment through voice distortion and triggers behavioural responses of the avatar as a response to the patient’s actions. Finally, VRAPT concludes with an evaluation of patients’ experiences, with particular emphasis on learning outcomes. The virtual environment used throughout all phases was rendered by the software Social Worlds (©CleVR).

Patients and therapists each have a workbook throughout the treatment, where they keep notes before and after each session. Both patients and therapists are expected to make notes on what to include in the next session and to evaluate the completed session. Thus, the workbooks become both a summary of, but also a tool for the treatment as a whole^1^.

## Ethical considerations

The present study, as part of a VRAPT pilot study, was approved by the Swedish Ethical Review Authority (Dnr: 2019–02337; 2020–06317). In such a population as the current sample, ethical considerations are of specific importance since forensic psychiatric patients are considered a particularly vulnerable group, showing wide-ranging needs of healthcare and societal interventions^12^. The inherent coerciveness of forensic settings^53^ accentuates the need for specific ethical considerations regarding, e.g., autonomy, deception, informed consent, mental liberty, moral agency and dignity^53–55^. Additional considerations should also be given to the risk of therapeutic misconception^1,56^, referring to misinterpretations or confusion regarding the difference between clinical research and ordinary treatment that may arise, potentially making participants feeling pressured to participate to progress in their care. Since the present study only analyzed pseudonymized data, some of the above-mentioned concerns might be seen as alleviated. Written informed consent was obtained from all participants following verbal and written explanations of both purpose and procedures involved as part of the pilot study. Likewise, information was given concerning the voluntary nature of participation and possibility of withdrawal of consent without the need for any explanation.

### Data sources

The study was conducted in accordance with guidelines for data saturation in qualitative studies^57^. Data from seven individual VRAPT treatments, reported in therapists’ and patients’ treatment workbooks (*N* = 7 + 6), at a maximum-security forensic psychiatric clinic were analyzed within this study, portraying how virtual reality-assisted aggression treatment was conducted in a clinical forensic psychiatric setting. This study follows the proposed saturation guidelines, which suggest 9–12 texts^57^. Even though not all treatment workbooks were returned, one patient declined to do so, the total data amounted to 13 texts. Thus, it would seem reasonable that data saturation was achieved. Overall, the treatment workbooks were structured session by session, the participant version spanning 126 pages and the therapist version 160 pages. Both versions described the ongoing and remaining work within the intervention, with space for participants’ own written reflections. Patients’ workbooks additionally included a section where each patient wrote down the homework for each session.

## Procedure and data analysis

All treatment workbooks were read several times by the main author (FS), a master’s student in clinical psychology with experience working in maximum-security forensic psychiatry, to facilitate an overall comprehension of the data. Thereafter, preliminary codes were assigned, and the data was further organized into content categories and subcategories. A content category describes the content at a manifest level, with a low degree of interpretation and a varying degree of abstraction^58^. The decision was made not to separately first read either patients’ or therapists’ workbooks, but rather to simultaneously read both, treatment by treatment. It was felt that this better facilitated a comprehensive understanding of each and every individual treatment, as well as of what, generally, had been the content of all seven treatments. All coding was initially conducted by the author (FS). However, final categories and subcategories were refined and determined in consensus with last author MW. Subsequently, these categories were interpreted, resulting in a concluding narrative synthesis. No software was used in the analysis process.

## Results

The following content categories were identified in the workbooks: Skills training, Tailoring of the intervention, and Self-awareness. An overview of the content categories and associated subcategories of patients’ and therapists’ perspectives on the revised VRAPT intervention is provided in Table 1, followed below by descriptions of each category and subcategory. Citations are followed by either P (Patient) or T (Therapist), together with the treatment number, within parentheses.

**Table 1:** *Content categories and subcategories of patients’ and therapists’ perspectives on the revised VRAPT intervention.*

Skills training	Tailoring of the intervention	Self-awareness
Relaxation techniques	Determining treatment goals	Early reactions to heightened aggression
Cognitive empathy	Adapting to participants’ needs and limitations	Awareness of own limitations
Interpersonal communication	Application in everyday life	What calms and helps

### Skills training

This content category stems from both patients’ and therapists’ perspectives on the acquisition of abilities to calm oneself, awareness and recognition of emotions, taking the perspectives of others, handling of conflicts and self-assertion, and finally training of different scenarios and role-play both in and outside of the VR environment. The following subcategories were identified: Relaxation techniques, Cognitive empathy, and Interpersonal communication.

#### Relaxation techniques

Evident from the treatment workbooks was how different ways of calming oneself were perceived as an integral and helpful part of the intervention. The value of being able to, in various ways, gain a sense of control over the situation, thus attaining perspective and reducing emotional arousal, was described as related to a lessening of aggressive impulses.

“*It was harder to be aggressive… used the breathing technique and then walked away*” (P2).

“*The breathing exercise worked fine*,* also being much appreciated by the participant*” (T5).

The mental act of considering the consequences of acting in one way or another, comparing anticipated results with one’s own internal goals was also perceived as a helpful part of the intervention.

“*I consider the consequences. Also*,* how to handle the situation in the best possible manner*” (P4).

“*Working on finding techniques that calms. Identifies “to consider the consequences” and “to consider my own goals*”” (T7).

One patient offered a slightly different perspective, highlighting the importance of learning from one’s mistakes.

“*Continue to know my own mistakes*,* and not repeating them even in difficult situations*” (P6).

#### Cognitive empathy

Patients described difficulties in awareness and recognition of emotions when confronted by the facial expressions of virtual avatars during the second phase of VRAPT. Taking the perspectives of others, and recognizing especially emotions of disgust and surprised/confused, was described as difficult.

“*The ability to discern the signals of others*,* I feel it’s more difficult with women but do not know why*” (P2).

“*When joking with another person I do not know when they feel ‘enough is enough’*” (P5).

To a substantial degree, these were seen to resonate with the perspective of therapists. The latter, however, while in agreement regarding disgust rather emphasized difficulties recognizing anger as opposed to surprised/confused. Furthermore, therapists also stressed how skills training in emotion recognition enabled the patient to better understand both him-/herself as well as those he/she interacted with.

“*Says that “recognizing emotions” has been rewarding It has made him think about how he is perceived. Realizes that there could be a difference in how one looks and how one feels and that there in between is a process called “interpretation”*” (T6).

#### Interpersonal communication

From the perspective of patients, the importance of attaining a sound self-assertiveness based on a sense of self-respect was clear. This, in turn, influenced aggressive impulses and was tied with the ability to verbally, as opposed to physically, tackle challenging situations.

“*Try and talk to the individual*,* like saying I understand that you are upset but you don’t need to be angry with me*” (P3).

“*-self-assertion even confronting through speaking your hearts intent not getting into an argument… also it’s not about getting people to adapt to what one feels it’s about self-respect*” (P4).

One patient elaborated on how the tackling of a difficult interpersonal situation was coupled with the ability to sometimes stay and speak one’s mind as opposed to withdraw from the situation. In that sense, interpersonal events could be described as a continuous negotiation of “space”.

“*To claim*,* or to give space*” (P4).

Therapists’ perspectives centered somewhat more towards establishing a joint understanding of the situation, exploring the other’s perspective and on different modes of communication.

“*Validate the other*,* formulate a shared problem*,* ask for help*” (T4).

“*I role-play different sides and ask him to sort these based on different modes of communication also how different goals are achieved through different styles. Pros and cons*” (T4).

“*To develop an alliance: ask questions*,* get the other to elaborate on what he/she is saying*,* let the other finish and remember “that they want what’s best for me”*” (T6).

### Tailoring of the intervention

As with the previous content category, this category stemmed from both patients’ and therapists’ perspectives, depicting their perception of identifying risk scenarios, on finding a basis for skills training, the appraisal of whether techniques and strategies actually were useful, goal-attainment, and finally on the adaptation of the intervention to the specific patient. The following subcategories were identified: Determining treatment goals, Adapting to participants’ needs and limitations, and Application in everyday life.

#### Determining treatment goal

Therapists’ perspectives accentuated the time and collaborative effort needed in defining risk scenarios and arriving at valid treatment goals. In most cases, ongoing dialogue was necessary throughout the intervention.

“*determining treatment goals and defining risk scenarios takes a long time. It felt to me and the participant that some things / questions were repetitive*” (T1).

From the perspective of therapists, it was clear how patients often had a hard time identifying what was difficult, and thus what should be the focus / goals of the treatment. Risk scenarios were often not merely being used as a foundation for role-play, but also in determining and reviewing overarching goals of the intervention.

“*“It’s hard to answer” (regarding what is difficult) even with follow-up questions the participant was unable to come up with anything*” (T3).

“*– the participant has a hard time coming up with new triggers*,* he says that he didn’t know before that he was sick but has now realized this*,* but doesn’t feel that there is all that much to practice on and that I am welcome to come up with scenarios and exercises of my own*” (T3).

Therapists could vary scenarios to gain new perspectives.

“*A surprise-scenario instead of the participant knowing in advance what was about to happen*” (T2).

“*Used certain scenarios as a surprise in order to get more of a response from the participant*” (T3).

Patients’ perceptions, on the other hand, seemed more to do with the ability to act constructively in challenging situations, a forward-looking perspective on furthering the attainment of life goals. Determining of treatment goals, for patients, thus seemed more focused on finding new ways of acting, with less focus on defining risks.

“*The ability to interpret one’s surroundings and not misconstrue situations. A lessening of suspicion towards unknown people*,* an outlook affected by the life I’ve lived*” (P2).

“*… to realize that the situation isn’t worth acting out or to wind up*,* suppress emotions that doesn’t lead to anything good for me or my future life*” (P2).

“*… being able to walk away from situations… to speak one’s mind in an acceptable manner… to explain to ward staff not to patronise me*,* rather talk to me in a pedagogic way*” (P3).

#### Adapting to patients’ needs and limitations

At the heart of therapists’ perspectives was the need to adapt to patients’ limitations, sometimes repeat and/or change scenarios multiple times and to role-play outside of the VR environment.

“*same scenario 3 times*,* not as strong reaction as the first time regardless of strategy*” (T2).

“*The participant says that these scenarios are what he needs and that they are much more realistic than previous ones…*” (T2).

However, role-playing realistic and, for the patient, triggering scenarios could become overwhelming why the focus of scenario enactment had to change.

“*The participant gets emotionally affected*,* tears up. Talking about his brother stirs anger and sadness. Focus a lot on behaviour… I role-play for him Works well!*” (T5).

Some patients described a preference for real-life role-plays instead of VR-assisted role-plays, as they experienced difficulties perceiving the VR milieu as authentic. Here, therapists adapted to patients’ preferences.

“*Experience VR as fake*,* and that it feels unnatural. Asks for role-play IRL*,* which we also do*” (T7).

Patients reported the need to feel a sense of familiarity with the situation at large, both in terms of the treatment and the therapist. One patient stated, in relation to both the overall treatment and the just concluded VR scenario, that time was needed in order to feel at ease.

“*After having gone through the same situation 2 times before*,* there was a familiarity to the situation and thus the 3 time it felt easier and less strenuous*” (P2).

#### Application in everyday life

Perspectives on the usefulness of techniques and strategies were mixed: on the one hand helpful in tackling situations reminiscent of the risk scenario, on the other hand sometimes hard to apply more generally.

“*– helpful in situations similar to the risk scenario*” (P3).

“*no situations on the ward where I could use the strategies*” (P3).

“*when angry and having to calm down and when tiered but having trouble sleeping*” (P5).

In contrast, one patient described the viewpoint of the techniques / strategies as altogether unhelpful.

“*Don’t feel it leads to any change*” (P7).

Nonetheless, therapists’ outlook on goal-attainment portrayed enhanced communicative capabilities, an insight into the effectiveness of relaxation techniques and emotional awareness.

“*– the participant realized the usefulness of communication- and relaxation strategies… generally much better at conversing where he previously had kept silent… learned it’s possible to mirror emotional states*” (T3).

On a similar note, one patient’s perspective also pointed to a better understanding of self and others, an awareness of own limitations and challenges.

“*–VRAPT has opened my eyes as to when I faulter in communicating and socialising with fellow humans –more knowledgeable of my shortcomings*,* wishing to better implement these methods in everyday life –has taught me being self-assertive is tough*,* since I never was previously in life*” (P4).

### Self-awareness

This content category, as perceived by patients, has to do with how the intervention clarified the meaning of physiological reactions, was illustrative of own limitations, together with what might be helpful. The following subcategories were discerned: Early reactions to heightened aggression, Awareness of own limitations, and What calms and helps.

#### Early reactions to heightened aggression

Several patients described a heightened awareness of how physiological, cognitive as well as behavioural changes act as cues to emotional arousal and elevated risk of aggressive behaviour.

“*increased heartbeat*,* sweating*,* gets ready to fight*” (P2).

“*Heart beats faster*,* more energy throughout the body*,* a sharpening of senses*” (P4).

“*I become terser*” (P6).

#### Awareness of own limitations

Patients described the importance of not getting caught up in old behavioural patterns; the difficulty of doing what one knows one should. One patient reported such awareness in response to a direct question in the treatment workbook regarding needed abilities and treatment goals.

“*to listen and not to argue*,* to speak my thoughts and feelings and recognize signs that I’m getting angry*” (P6).

However, another patient summed up a general theme of although having gone through the intervention and being more aware of own triggers and responses, it nonetheless could be difficult to act constructively in a challenging situation.

“*When in conflict with somebody it can be hard to walk away*” (P5).

#### What calms and helps

Patients’ perspectives considered attaining control of oneself and the situation, of finding ways of giving oneself time to pause and reflect, as well as how this was practiced time and time again throughout the intervention.

“*Breath in calmly through the nose and breath out through the mouth*” (P3).

Specifically, the helpfulness of a physical distance to other people, and the importance of being left alone, was emphasized.

“*…having a distance to people*” (P3).

However, some patients elaborated on that merely distance and control was not enough, instead stressing the need for distraction. Distraction was reported as a prerequisite for the lessening of emotional arousal and therethrough the sense of being able to handle the situation.

“*Not merely controlling the anger*,* but also deceiving myself with methods of distraction*” (P2).

“*Do something relaxing… listen to music*,* exercise*” (P7).

## Discussion

The principal focus of this study was to highlight the perspectives of patients and therapists regarding the treatment content of the revised VRAPT intervention, and therethrough implicitly also addressing how VRAPT was clinically implemented in a high-security forensic psychiatric setting. Of particular interest for present purposes was how the perspectives of patients and therapists on the actual treatment content of VRAPT were seen to not merely converge but occasionally also differ in potentially important ways, e.g. as pertains to the determination of treatment goals, interpersonal communication, and the underlying reasons for the need for adaptations of the intervention. In sum, three content categories were manifest in the data: Skills training, Tailoring of the intervention, and Self-awareness.

### Skills training

Overall, the present findings suggest that skills training during VRAPT principally comprised three separate parts: the awareness and recognition of emotions depicted in the VR environment, techniques to calm oneself, and finally the enactment of scenarios and role-play both within and without of the virtual milieu. However, combining patients’ and therapists’ perspectives, it became evident how these, albeit being separate parts, nonetheless were perceived as a coherent treatment in large focused on interpersonal communication. Accordingly, patients’ perspectives seemed to a substantial degree to resonate towards how awareness and recognition, as well as the communication of emotional states, enabled them to establish and maintain a sense of self-respect. One patient even pointed out that it is not so much to get others to change according to how one feels, but rather that being able to recognize emotions in oneself and others is a piece of the puzzle that enables a sound self-assertiveness. Here, the inherent coerciveness in forensic psychiatry^53–55^. Patients also emphasized difficulties in discerning emotional signals from others. However, patients and therapists reported different emotions as the most difficult for patients to recognize; patients described disgust, together with surprised / confused, as the most difficult whereas therapists aligned with patients on disgust but also stressed difficulties recognizing anger. The current study is insufficient for conclusions as to whether this may signal a genuine lack of ability, as has been proposed in previous research on forensic psychiatric patients^59,60^ or conversely be better appropriated to issues with the rendering of the facial expressions in the virtual environment. Returning to the focus of interpersonal communication, therapists offered a slightly different perspective in accentuating the establishment of a shared understanding of any given interpersonal event, to formulate a mutual problem and to invite the other to elaborate on his/her view. Thus, it would seem that therapists to a higher degree forwarded working on different modes of communication, to consider the wishes of others as well as the need to adapt to one’s surroundings as central aspects of the intervention.

Emergent from both perspectives was how the attainment of ways to calm oneself was at the heart of VRAPT, but also how a collaborative effort was both needed and helpful in arriving at the precise techniques that were suitable for the specific patient. Moreover, how the intervention not merely provided suggestions for new ways to remain calm in challenging situations, but how it also facilitated a joint elaboration on already present abilities of the patient, hereby furthering participation. All the same, taking the reasoning a bit further, perhaps the helpfulness of having ways to calm oneself could be best understood as a means to an end; especially patients highlighted how the ability to stay calm provided them with a sense of control of the situation. Thus, it could be argued that the treatment, while focusing on techniques to calm down, lay a foundation for the attainment of perspective and thereby freeing the patient to act in less aggressive ways through feeling less vulnerable.

Patients as well as therapists recognized role-plays, regardless of whether it was conducted inside or outside of the VR environment, as the principal means through which other aspects of the treatment intervention were brought into play. Role-plays provided a changeable interpersonal event and constituted a needed arena for a safe exploration of alternative roles, reminiscent of the notion of psychological treatment involving an essential component of play^61^. Here, the importance of development of a therapeutic alliance can be seen through using role-plays as an ongoing collaboration as well as adaptation of the treatment content to patients’ needs^62,63^.

### Tailoring of the intervention

Therapists reported difficulties for patients to present risk scenarios and aggression-triggering events, demonstrating the substantial time and effort that was needed in order to formulate viable treatment goals and the corresponding role-play scenarios. Continuously maintaining a dialogue back and forth was portrayed as crucial for the ongoing adaptation to patients’ needs and limitations. Here, patients favored a forward-looking perspective where they focused on finding adaptive ways of tackling challenging situations. Therapists, on their hand, focused on identification of risks and finding concrete situational cues to what might pose a challenge and how this in turn could be utilized in the enactment of scenarios in VR. Taken together, the patients seemingly favored a recovery-oriented approach while therapists seemed to favor a risk-oriented approach^64^. While this may seem as different approaches, a common denominator is how a collaborative effort in constructing role-play scenarios is emphasized and, thus, what aspects should be the focus of the treatment. Finding transparent ways of collaborating between patients and therapists and staff in forensic psychiatry is at the core of recent studies on patient participation in high-security forensic psychiatric care^65^.

### Self-awareness

From patients’ notes in treatment workbooks, it became clear how their own reflections, little by little, took the form of a progression towards an openness to try out new ways of thinking and acting during the course of treatment. While many factors affect the process of any aggression treatment, a common denominator is an increased capacity for, and motivation to, engage in self-regulation of thoughts and feelings and the interconnectedness between them^3^. Longitudinal research provides support for significant linkages between self-perceived low regulatory efficacy and patterns of aggression and aggressive behaviour^66–68^. A study^69^ found a connection between poor self-regulation, off-course attempts at conflict management, inability to harbour negative emotions, and aggressive recidivism in a sample of adult reoffenders. Thus, it would seem that the treatment content of repeatedly confronting the patient with ever-changing interpersonal events through role-play could engender a qualitative shift in some patients’ ability to both recognize and utilize physiological, cognitive and emotional signs of heightened aggression in the handling of challenging situations.

Evident was also, from the perspective of patients, how the treatment for some had fostered a change towards a recognition of the necessary balancing act of on the one hand standing up for what one believes in, to establish and maintain self-respect, and on the other expressing themselves in socially acceptable ways. Or, perhaps one patient said it best: “To claim, or to give space” (P4).

In sum, and perhaps unsurprisingly, within both perspectives lies the notion that reduction in aggression is engendered through combinations of the different parts of the intervention. Hereby, arguably, some support is offered for the need of integrative frameworks in any properly articulated theory of aggression treatment^3^.

### Limitations and strengths

One of the primary limitations of this study concerns the inherent coerciveness of forensic psychiatric settings, which may have influenced participant responses and engagement^70^. The therapeutic misconception, where patients fail to distinguish between participation in research and routine clinical care, poses a significant ethical and methodological challenge^56^. Patients may feel obligated to participate, believing that their engagement in VRAPT might influence their treatment trajectory or legal status^27^. Moreover, some may have misinterpreted research participation as a requirement rather than a voluntary decision, either due to miscommunication or personal assumptions. To mitigate this risk, this study implemented a comprehensive informed consent procedure, including detailed verbal and written explanations emphasizing the voluntary nature of participation. Despite these efforts, the possibility of subtle coercion cannot be entirely ruled out. Some evidence suggests that participants retained their autonomy, such as one patient declining to return their workbook and others preferring face-to-face role-play over VR-assisted sessions, indicating an ability to make independent treatment-related choices. However, future research must further refine communication strategies to prevent misconceptions about participation.

Another limitation relates to the gender imbalance within the sample. Of the seven participants, six were male, which reflects the general demographic composition of forensic psychiatric populations, where male patients significantly outnumber female patients^12^. Nevertheless, this skewed representation limits the generalizability of findings to female patients, as gender may influence aggression patterns, emotional regulation, and responsiveness to treatment^16^. Future studies should either strive for a more balanced sample or investigate gender-specific adaptations of VRAPT to assess potential differences in treatment effectiveness.

A methodological constraint of this study is its reliance on workbook-based data collection, which, while offering structured insights from both patients and therapists, introduces several concerns. Workbooks depend on self-reported reflections, making the data vulnerable to recall bias, social desirability bias, and response distortion^71^. Patients, particularly in forensic psychiatric settings, may underreport negative experiences or exaggerate their progress due to institutional pressures or the belief that participation might positively impact their treatment evaluations^56^. Similarly, therapists may unconsciously frame their reflections in alignment with expected therapeutic goals, rather than providing entirely objective evaluations^72^. Additionally, inconsistent engagement with workbooks across participants presents another challenge. Some participants provided rich, detailed reflections, while others contributed minimal responses or omitted sections entirely, creating gaps in data. Factors such as literacy levels, cognitive impairments, or motivation may have affected participants’ ability to engage with workbook tasks^73^. This variability jeopardizes data saturation, as themes may be overrepresented or underrepresented depending on which participants provided the most comprehensive reflections^57^.

One notable limitation concerns the reliability of data processing. While coding was initially performed by one researcher and subsequently reviewed by a second author, formal inter-rater reliability measures were not calculated. Although this process provided a degree of validation, the absence of quantified inter-rater agreement may impact the replicability of the findings. Furthermore, the workbooks used in the intervention, although described in detail and tailored to the specific aims of VRAPT, have not been formally tested for psychometric reliability and are not based on previously validated instruments. Future research should seek to strengthen methodological rigor by incorporating inter-rater reliability assessments and evaluating the reliability and validity of intervention materials.Furthermore, workbooks capture post-session reflections rather than real-time experiences, leading to potential memory distortions. This is particularly relevant in emotionally intense interventions like VRAPT, where patients may recall only the most salient or emotionally charged moments, rather than providing a comprehensive account of each session^74^. Future research should explore real-time data collection methods to complement self-report measures.

Despite these limitations, this study offers several notable strengths. A key advantage is its dual-perspective approach, incorporating insights from both patients and therapists who participated in the same VRAPT intervention. This methodological approach enhances the depth of analysis, providing a more comprehensive understanding of the treatment experience and implementation challenges. Additionally, by focusing on an innovative aggression treatment method utilizing VR technology, this study contributes to the growing body of research exploring novel interventions in forensic psychiatry^1^.

To our knowledge, no previous studies have specifically analyzed the content or use of the workbooks in the context of the VRAPT treatment. While earlier VR studies have focused on outcomes such as emotional regulation, aggression reduction, or user experience, the integration and role of structured therapeutic materials, such as the VRAPT workbooks, have not been the subject of systematic investigation. This study therefore provides a novel contribution by shedding light on how these materials are perceived and used by participants within a forensic psychiatric setting.

Another relevant limitation concerns the potential influence of social desirability in patient reports. Given the clinical and institutional context in which the study was conducted, participants may have felt inclined to present themselves in a favorable light or to provide responses they believed were expected by staff or researchers. This tendency could have influenced the content of the workbook entries as well as verbal feedback during interviews, thereby impacting the reliability of the data. While efforts were made to create a safe and non-judgmental environment, the possibility of socially desirable responding should be considered when interpreting the findings.

The findings highlight both potential benefits and areas for improvement, paving the way for future refinement to VRAPT. Furthermore, this study underscores the importance of interpersonal communication skills in aggression treatment. While previous research has emphasized emotional regulation as a primary mechanism of change, this study suggests that enhancing self-assertiveness and emotional awareness may be equally critical in reducing aggression^73^. Future VR-assisted treatments should further integrate interpersonal communication training to maximize therapeutic outcomes.

### Recommendations for future research

To enhance data validity and depth, future studies should incorporate complementary methodologies that reduce self-report biases and provide a more objective assessment of VRAPT’s effectiveness. Potential approaches could include: recording VRAPT sessions would allow researchers to objectively assess behavioral and emotional responses during treatment, direct therapist observations could provide a more nuanced evaluation of patient engagement and progress^75^. Using heart rate variability (HRV), electrodermal activity (EDA), and skin conductance can help track physiological responses and provide objective measures of emotional engagement during VRAPT sessions^74^. These measures could help identify patterns of emotional regulation, particularly in response to high-intensity VR scenarios. Eye-tracking technology can analyze participants’ attentional focus and emotional engagement during VRAPT scenarios^76^. Motion analysis can track physical reactions and engagement levels in VR environments, offering valuable data on patient responses. Follow-up interviews with patients and therapists, conducting structured post-treatment interviews would allow researchers to clarify ambiguous workbook reflections and explore patient experiences in greater depth^77^. This approach would triangulate qualitative data, reducing the risk of interpretation bias. Finally, future developments of the VRAPT could integrate the GAM model more clearly, and investigate this in relation to treatment outcome.

## Conclusions

The findings of this study provide critical insights into the treatment content and clinical implementation of the revised Virtual Reality Aggression Prevention Training (VRAPT) intervention in forensic psychiatry. The collaborative nature of the intervention was consistently emphasized by both patients and therapists, suggesting that VRAPT fosters openness to new ways of interacting and enhances self-awareness. However, notable differences in perspectives emerged between the two groups. Therapists primarily focused on identifying triggers of aggression (risk-perspective), while patients placed greater emphasis on strategies for managing aggression effectively (recovery-perspective). This discrepancy underscores the importance of bridging these viewpoints to ensure a more holistic and patient-centered approach in aggression treatment.

Furthermore, role-play emerged as a central component of VRAPT, yet it was not merely a technique for behavioral rehearsal; rather, it served to enhance interpersonal abilities. While both therapists and patients recognized its importance, patients particularly associated role-play with self-respect and self-assertiveness, suggesting that the intervention’s effectiveness may extend beyond aggression management to broader social and emotional regulation skills. From a practical standpoint, the intervention appears to focus more on highlighting patient limitations rather than reinforcing existing strengths, which may impact engagement and perceived treatment efficacy. To maximize therapeutic benefits, a more integrated approach that balances risk-awareness with patient empowerment may be beneficial. Aligning the forward-looking, solution-focused approach of patients with the risk-oriented framework of therapists could further enhance treatment outcomes and facilitate VRAPT’s successful integration into routine forensic psychiatric care.

While this study has certain methodological limitations, including coercion risks, self-report bias, and gender imbalances, it nonetheless represents a valuable contribution to forensic psychiatric research. By leveraging technological advancements such as physiological monitoring, digital tracking, and structured post-session interviews, future research can enhance the validity of VR-assisted aggression interventions. These innovations will not only improve data reliability and patient engagement but also optimize VRAPT’s applicability in clinical settings, ensuring its effectiveness in real-world forensic psychiatric practice.

## Data Availability

Data cannot be shared openly but are available on request from authors. If the authors would like to request data from this study, author MW should be contacted.
